# Supplementary material for: Training enhances the ability of listeners to exploit visual information for auditory scene analysis
Source: Cognition. 2021 Mar;208:104529. doi: 10.1016/j.cognition.2020.104529 (PMC7868888; doi:10.1016/j.cognition.2020.104529)
Supplement: Supplemental Table 1 — The timing of the auditory/visual “blips” for each of the three demo files. The target vowel in the target coherent condition is /u/, whereas in the masker coherent and neither coherent conditions /a/ is the target vowel. In the actual experiment the presentation of all pitches and timbres were counterbalanced. The size of the timbre “blip” is larger than in the actual experiment in order to make it more salient for demonstration purposes. [file mmc5.docx]

| Condition | Target Blip times (s) | Masker blip times (s) | Visual blip times (s) |
| --- | --- | --- | --- |
| Target Coherent | 4.40  10.16 | 3.00  6.76  11.25 | 13.70 |
| Masker Coherent | 4.86  8.38 | 6.49 | 10.26 |
| Neither coherent | 5.34  13.45 | 6.78  8.07 | 3.00 |

**Supplemental Table 1**

The timing of the auditory/visual “blips” for each of the three demo files. The target vowel in the target coherent condition is /u/, whereas in the masker coherent and neither coherent conditions /a/ is the target vowel. In the actual experiment the presentation of all pitches and timbres were counterbalanced. The size of the timbre “blip” is larger than in the actual experiment in order to make it more salient for demonstration purposes.
